# Ectopic FOXP3 Expression in Combination with TGF-β1 and IL-2 Stimulation Generates Limited Suppressive Function in Human Primary Activated Thymocytes Ex Vivo

**DOI:** 10.3390/biomedicines9050461

**Published:** 2021-04-23

**Authors:** Jorge Gallego-Valle, Sergio Gil-Manso, Ana Pita, Esther Bernaldo-de-Quirós, Rocío López-Esteban, Marta Martínez-Bonet, Verónica Astrid Pérez-Fernández, Ramón Pérez-Caballero, Carlos Pardo, Juan-Miguel Gil-Jaurena, Rafael Correa-Rocha, Marjorie Pion

**Affiliations:** 1 Laboratory of Immune-Regulation, Gregorio Marañón Health Research Institute (IiSGM), Hospital General Gregorio Marañon, c/Dr. Esquerdo, 46, 28007 Madrid, Spain; jorge.gallego@iisgm.com (J.G.-V.); sergio.gil.277@hotmail.es (S.G.-M.); esther.bernaldo@iisgm.com (E.B.-d.-Q.); rocio.lopez@iisgm.com (R.L.-E.); marta.mbonet@iisgm.com (M.M.-B.); veronica.perez@iisgm.com (V.A.P.-F.); rafael.correa@iisgm.com (R.C.-R.); 2 Pediatric Cardiac Surgery Unit (HGUGM), Hospital Materno Infantil del Hospital General Universitario Gregorio Marañón, c/O’Donnell, 48, 28009 Madrid, Spain; anamaria.pita@salud.madrid.org (A.P.); ramon.perez-caballero@salud.madrid.org (R.P.-C.); carlosandres.pardo@salud.madrid.org (C.P.); juanmiguel.gil@salud.madrid.org (J.-M.G.-J.)

**Keywords:** human thymocytes, regulatory T cells, FOXP3, engineering cells

## Abstract

Regulatory T cells (Tregs), which are characterized by the expression of the transcription factor forkhead box P3 (FOXP3), are the main immune cells that induce tolerance and are regulators of immune homeostasis. Natural Treg cells (nTregs), described as CD4^+^CD25^+^FOXP3^+^, are generated in the thymus via activation and cytokine signaling. Transforming growth factor beta type 1 (TGF-β1) is pivotal to the generation of the nTreg lineage, its maintenance in the thymus, and to generating induced Treg cells (iTregs) in the periphery or in vitro arising from conventional T cells (Tconvs). Here, we tested whether TGF-β1 treatment, associated with interleukin-2 (IL-2) and CD3/CD28 stimulation, could generate functional Treg-like cells from human thymocytes in vitro, as it does from Tconvs. Additionally, we genetically manipulated the cells for ectopic FOXP3 expression, along with the TGF-β1 treatment. We demonstrated that TGF-β1 and ectopic FOXP3, combined with IL-2 and through CD3/CD28 activation, transformed human thymocytes into cells that expressed high levels of Treg-associated markers. However, these cells also presented a lack of homogeneous suppressive function and an unstable proinflammatory cytokine profile. Therefore, thymocyte-derived cells, activated with the same stimuli as Tconvs, were not an appropriate alternative for inducing cells with a Treg-like phenotype and function.

## 1. Introduction

Natural regulatory T cells (nTregs), derived from the thymus, are known to be essential for maintaining immune homeostasis, inducing tolerance, preventing inappropriate responses to commensal organisms, and dampening effector T cell responses following immune activation [1]. A reduction in their absolute number or frequency or an impairment of their function can trigger autoimmune, infectious, or allergy-associated diseases [2]. Due to their ability to control dysregulated immune responses, Tregs from the periphery have been used in clinical trials of patients with autoimmune diseases [3,4,5] or graft-versus-host diseases [6]. These trials have shown that Treg infusions are safe but rarely successful. This low success rate might be attributed to the fact that nTregs make up less than 10% of the total CD4^+^ T helper cells in the peripheral blood circulation and must be expanded in vitro to obtain the number of cells needed to treat a patient [7]. This leads to another limitation: this expansion worsens the functional efficiency of the nTregs [8], which rapidly become exhausted and therefore exhibit a reduced suppressive function. To overcome the issues of the limited number of nTregs that can be isolated from the periphery and their subsequent expansion, conventional CD4^+^ T cells (Tconvs) from peripheral blood have been stimulated in the presence of transforming growth factor beta type 1 (TGF-β-1) [8,9,10,11] or genetically modified to express exogenous FOXP3 to induce functional iTregs [12,13,14]. This latter approach generated many Tregs with stable FOXP3 expression, which could not be controlled by FOXP3 promoter regions and their epigenetic modifications, ensuring their stability [15,16].

Despite the above-mentioned advances, iTregs exhibit cellular plasticity [17,18]. Peripheral nTregs or iTregs derived from Tconvs can lose their FOXP3 expression—acquiring a CD4^+^ memory effector phenotype—and therefore produce IL-17A under proinflammatory microenvironments [19,20,21]. Therefore, even though these approaches generate high numbers of functional iTregs, they still present several limitations, such as age-associated Treg cellular exhaustion [22] and cellular instability [23]. The Treg lineage appears in the pediatric thymus, from which it migrates to the periphery. Recently, Dijke et al. presented the pediatric thymus as an alternative source of therapeutic naïve thymic Treg cells (tTregs) [24]. There are two types of Treg progenitors found in the thymus: CD25^+^FOXP3^neg^ and CD25^neg^FOXP3^low^. Both progenitors can differentiate into Tregs (CD25^+^FOXP3^+^) under IL-2 stimulation in vivo and in vitro [25]. The thymus microenvironment guarantees sustained FOXP3 expression, which generates a broadly functional nTreg repertoire from Treg progenitors [26,27]. TGF-β1 is essential in this process [8], as are other factors, such as IL-7 or IL-15 [28,29]. Human tTregs present remarkable purity, high epigenetic stability, and a robust suppressive function [24,30]. Moreover, their phenotype is highly naïve, and it has been observed that induced Tregs from the periphery had higher stability and functionality when generated from naïve cells instead of the total Tconvs from peripheral blood [3,21,31]. Therefore, tTregs should be highly relevant in future immunotherapies.

However, tTregs comprise around 1% of the total cells from the thymus [25]. Therefore, we tested if a high percentage of the CD25-negative fraction (CD25^neg^) of primary human thymocytes could be induced toward functional Tregs in vitro using TGF-β1 stimulation and the ectopic expression of the FOXP3 transcription factor, since both elements have been previously described to generate iTregs from peripheral Tconvs. First, we demonstrated that a CD25^neg^ subset stimulated by IL-2, TGF-β1, and CD3/CD28 could express high Treg-associated markers but showed a lack of a homogeneous suppressive function. Then, FOXP3-transduced CD25^neg^CD8^neg^ thymocytes, along with TGF-β1 stimulation, were also able to express a high level of FOXP3 without reaching the functionality of nTregs. In addition, in all the stimulated conditions, the exogenous FOXP3 expression and Treg-associated markers in cells were reduced as the culture time increased, and cells produced proinflammatory cytokines showing a differential profile regarding nTregs. These results indicated that TGF-β1 stimulation and high FOXP3 expression might not be sufficient to convert undifferentiated thymocytes into iTregs. Hence, thymic CD25^neg^ cells do not constitute a viable alternative source of iTregs under these culture conditions.

## 2. Methods

### 2.1. Patient Samples

Pediatric thymic tissues were obtained from 18 children (13 males and 5 females) from 4 days to 6.3 years old (mean = 385.9 days; median = 138 days). Written, informed consent for study participation was obtained from the parents or guardians of all the individuals. The Pediatric Cardiology Unit enrolled all the pediatric individuals under a protocol accepted by the clinical ethics committee of the Gregorio Marañón Hospital (reference number THYTECH1-2018-005/CIRSCAR-ThyTREG18) according to the principles described in the Declaration of Helsinki (2013). These patients were undergoing corrective cardiac surgery due to different heart pathologies in the Infantile Cardiac Surgery Unit at Gregorio Marañón General University Hospital. The entire thymus is routinely removed to release the surgical field during cardiac surgery, and it is discarded. The children had no known immunological or genetic abnormalities.

### 2.2. Regulatory T Cell Isolation from Thymic Tissue

Thymocytes were obtained by the mechanic disruption of the thymus tissue from pediatric patients using a gentleMACS dissociator (Miltenyi Biotec, Bergisch Gladbach, Germany). CD25^+^ regulatory T cells (CD25^+^) and CD25-negative T cells (CD25^neg^) were purified using CD25 Microbeads (Miltenyi Biotec, Bergisch Gladbach, Germany) from the total thymocytes. The purity of the CD25^+^ was better than 70%, and that of CD25^neg^ was better than 95% (74.29 ± 2.52% and 96.69 ± 0.43%, mean ± SEM, respectively; S4 in Supplementary Materials). From the CD25^neg^ fraction, the CD25^neg^CD8^+^ and CD25^neg^CD8^neg^ fractions were isolated using CD8 Microbeads (Miltenyi Biotec, Bergisch Gladbach, Germany), according to the manufacturer’s protocol. CD25^+^ and all the CD25^neg^ fractions were cultured with an X-vivo15 medium (Lonza, Walkersville, MD, USA) supplemented with 5% AB human serum (Sigma-Aldrich, St. Louis, MO, USA) and a 1% mix of antibiotics (125 µg/mL ampicillin, 125 µg/mL cloxacillin, and 40 µg/mL gentamicin, Sigma-Aldrich, St. Louis, MO, USA).

### 2.3. Isolation of Peripheral Naïve nTregs

Naïve CD4^+^CD25^neg^ (Tconvs) and naïve CD4^+^CD25^+^ T cells (nTregs) were freshly isolated from buffy coats using a CD4^+^CD25^+^CD45RA^+^ Regulatory T Cell Isolation Kit according to the manufacturer’s instructions (Miltenyi Biotech Bergisch Gladbach, Germany). The cells were cultured with an X-vivo15 medium supplemented with 5% AB human serum and a 1% mix of antibiotics (125 µg/mL ampicillin, 125 µg/mL cloxacillin, and 40 µg/mL gentamicin).

### 2.4. Lentiviral Vector Production

The psPAX2 and pMD2.G plasmids (both plasmids were a gift from Prof. Dr. Trono, Addgene, Watertown, MA, USA) were used with pMSCV-FOXP3-EF1α-GFP-T2A-Puro (MSCV-FOXP3, SBI System Biosciences, Palo Alto, CA, USA) or the same plasmid without the FOXP3 sequence (MSCV-ΔFOXP3). MSCV-ΔFOXP3 was created using the restriction enzymes EcoRI and NotI from the original MSCV-FOXP3 sequence, and it was used as the negative control. Lentiviral vectors were produced by the co-transfection of 293T cells (ATCC, LGC Standards S.L.U., Barcelona, Spain) with MSCV-FOXP3 (or MSCV-ΔFOXP3), psPAX2, and pMD2.G using a calcium phosphate transfection kit (Sigma-Aldrich, St. Louis, MO, USA). The physical titers of the vectors were evaluated after 0.45 µm filtration (Corning, Corning, NY, USA) by quantifying HIV-1-p24^gag^ with an ELISA kit (Abcam, Cambridge, UK). The MSCV-FOXP3 and MSCV-ΔFOXP3 vectors both encoded GFP (the transduced cells could be assessed by flow cytometry) and a selectable marker for puromycin resistance.

### 2.5. Cell Culture of TGF-β1-Stimulated Cells

Natural Treg cells, peripheral naïve CD25^neg^ cells, thymus-derived CD25^+^, and CD25^neg^ cells were stimulated with Dynabeads Human T-Activator CD3/CD28 (Dynabeads, Life Technologies AS, Norway) for 72 h. Then, the Dynabeads were removed, and the cells were cultured for another 7 days. Throughout this process, the cells were cultured with 600 U/mL of IL-2 (ImmunoTools, Friesoythe, Germany), and half of the CD25^neg^ cells were stimulated with TGF-β1 (10 ng/mL, ImmunoTools, Friesoythe, Germany). The culture medium and cytokines were replaced every two days. The CD25^+^ cells were used as a positive control for the Treg phenotype and functional and stability assays [24].

### 2.6. Cell Culture of FOXP3-Transduced Stimulated Cells

CD25^+^ and CD25^neg^CD8^neg^ cells were stimulated with Dynabeads Human T-Activator CD3/CD28. Then, 24 h after activation, the CD25^neg^CD8^neg^ cells were transduced with lentiviral vectors at a multiplicity of infection of two, resulting in two CD25^neg^CD8^neg^ conditions (i.e., CD25^neg^CD8^neg^FOXP3 and CD25^neg^CD8^neg^ΔFOXP3). Then, 72 h post-activation, the Dynabeads were removed, and the cells were extensively washed and then cultured with puromycin (2.5 µg/mL, Sigma-Aldrich, St. Louis, MO, USA) for another 3 days. Throughout this process the cells were cultured with 600 U/mL of IL-2 and TGF-β1 (10 ng/mL). The culture medium and cytokines were replaced every two days.

### 2.7. Phenotype Analysis by Flow Cytometry and FOXP3 Western Blotting

The transduced cells were assessed on Days 3, 7, and 14 for GFP expression and viability using 7-aminoactinomycin D (7AAD, Sigma-Aldrich, St. Louis, MO, USA). FOXP3-transduced and TGF-β1-stimulated cells were stained on Days 0, 3, 7, and 10 or 14 post-culture to define their phenotypes, assessing their surface and intracellular markers by flow cytometry. Briefly, the cells were surface stained and stained with Fixable Viability Dye-eFluor450 (eBioscience, San Diego, CA, USA) to differentiate living and dead cells. Then, the cells were fixed or permeabilized using a FOXP3 transcription factor staining kit (eBioscience, San Diego, CA, USA) for intracellular staining. All the antibodies are listed in Table S1. Then, the cells were analyzed by flow cytometry using a Gallios cytometer (Beckman Coulter, Nyon, Switzerland), and the data were analyzed using Kaluza software (Beckman Coulter, Nyon, Switzerland). Protein localization was assessed by western blots using anti-human Foxp3 mAb PCH101 antibodies (eBioscience, San Diego, CA, USA), Actin antibodies Clone AC-40 (Sigma-Aldrich, St. Louis, MO, USA), and Lamin Monoclonal Ab mab636 (ThermoFisher, Waltham, MA, USA).

### 2.8. Proliferation and Suppression Assay and Stability of the Cells in the Suppression Assay

Allogeneic CellTrace Violet (CTVio, Life Technologies, Carlsbad, CA, USA)-labeled PBMCs were co-cultured with CD25^neg^ fraction (both with and without TGF-β1 treatment), CD25^neg^CD8^neg^ (transduced or non-transduced conditions and with TGF-β1 stimulation), or CD25^+^ cells at ratios of 2:1, 1:1, and 1:2 of suppressor cells/CTVio target cells, respectively. Each condition except the negative control of proliferation was stimulated with Dynabeads (Life Technologies) supplemented with 60 U/mL of IL-2 (ImmunoTools, Friesoythe, Germany). After 72 h, the cells were surface stained and stained with 0.5 µg/mL of 7AAD to differentiate living and dead cells. All the antibodies are listed in Table S1. Using the CTVio signals, the proliferation of the allogeneic CD4^+^ T cells was analyzed by assessing the reduction in the proliferation dye (CTVio) by flow cytometry. To calculate the percentage of suppression of proliferation, the division index method was used as previously described in [32]. To determine the phenotypic stability of the thymocyte-derived cells used in the suppressive assay, the cells were analyzed before and after the assay. The cells were surface stained and stained with Fixable Viability Dye-eFluor450 (eBioscience, San Diego, CA, USA) to differentiate living and dead cells, and then the cells were fixed or permeabilized using the FOXP3 transcription factor staining kit for intracellular staining. All the antibodies are listed in Table S1.

### 2.9. Stability Assay in the Proinflammatory Environment for TGF-β1-Stimulated Cells

On Day 7, the phenotypic stability of CD25^neg^ (with or without TGF-β1 stimulation) and CD25^+^ cells was evaluated by culturing them under proinflammatory conditions. Each cell subset was tested with Th1 and Th17 polarizing conditions, using IL-12 (10 ng/mL) for the Th1 and IL-1β, IL-6, IL-23 (10 ng/mL each), and tumor necrosis factor (TNF)-α (20 ng/mL) for Th17 (all cytokines were from ImmunoTools, Friesoythe, Germany) [33]. Moreover, a non-treated (NT) condition for each cell subset without proinflammatory cytokines was also established as the negative control. The cells were restimulated with Dynabeads supplemented with 600 U/mL of IL-2. After 72 h, the supernatant was collected, and the Dynabeads were removed. The cells were cultured again with the corresponding cytokines for an additional 72 h. After this culture time, the cells were stimulated with Phorbol 12-myristate 13-acetate (PMA, 50 ng/mL, Sigma-Aldrich, St. Louis, MO, USA) + Ionomycin (1 µg/mL, Sigma-Aldrich, St. Louis, MO, USA) for 5.5 h and brefeldin (Sigma-Aldrich, St. Louis, MO, USA) during the last 2 h of incubation. Then, the cells were washed, surface stained, stained for viable cells with Fixable Viability Dye eFluor450, fixed or permeabilized (FOXP3 transcription factor staining kit, eBioscience, San Diego, CA, USA), and stained for intracellular cytokines. All the antibodies are listed in Table S1.

### 2.10. Anti-Inflammatory and Proinflammatory Profile Analysis on FOXP3-Transduced Cells

To determine their cytokine production, on Days 7 and 14, the transduced CD25^neg^CD8^neg^ were stimulated with PMA (50 ng/mL) + Ionomycin (1 µg/mL) for 5.5 h and brefeldin A (10 µg/mL) during the last 2 h of incubation. Then, the cells were washed, surface stained, stained for viability with Fixable Viability Dye eFluor450, fixed or permeabilized (FOXP3 transcription factor staining kit, eBioscience, San Diego, CA, USA), and stained for intracellular cytokines. All the antibodies are listed in Table S1.

## 3. Statistical Analysis

The results are expressed as the mean ± SEM. The statistical comparisons of the phenotype and of the suppressive function by the reduction in the CTVio signal between CD25^neg^ (treated or non-treated with TGF-β1), CD25^neg^CD8^neg^, and CD25^+^ were performed using one-way ANOVA or two-way ANOVA followed by Bonferroni correction for multiple tests. The expression markers’ statistical comparisons before and after the suppression assay were performed using the nonparametric Mann–Whitney U test. The statistical associations between variables were calculated by linear regression and Spearman rank correlation analysis, where *p*-values < 0.05 were considered to be statistically significant. All the analyses and graphical displays were performed with GraphPad Prism (v.7.00 for Windows, GraphPad Software, La Jolla, CA, USA).

## 4. Results

### 4.1. Viability and Phenotype of Thymocyte-Derived Cells

To study the possibility of generating human Tregs from thymocytes, we first collected thymocytes from the thymus and then isolated CD25^+^ and CD25^neg^ fractions. As CD25^+^ thymocytes from the thymus have previously been described as tTregs, this fraction was considered to be our positive control for studying Treg phenotype, function, and stability [25].

**Figure 1:**
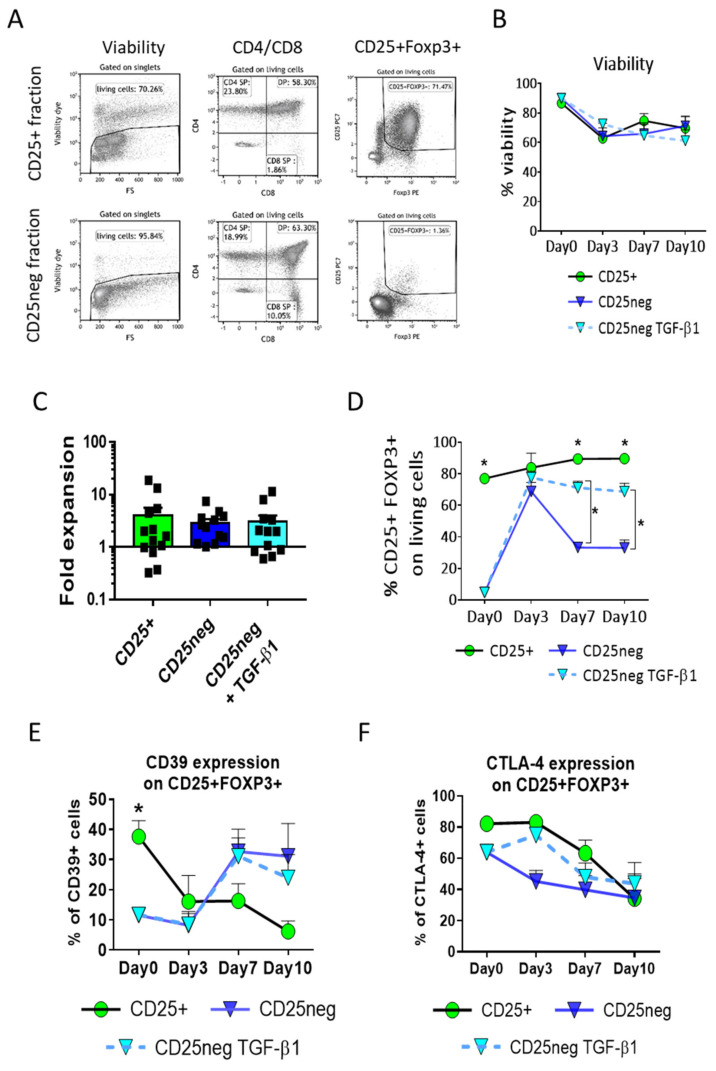
Viability and phenotype after cell isolation and throughout the cell culture. CD25^+^ and CD25-negative (CD25^neg^) fractions are the positive and negative fractions of CD25^+^ selection from the thymocytes. The viability and phenotype of each fraction were analyzed by flow cytometry. (**A**) Dot plots represent the percentage of viability, CD4/CD8 subsets, and CD25/FOXP3 surface labeling of each fraction on the day of cell isolation. Dot plots belong to one representative experiment from 14 samples. (CD4SP, CD4^+^ simple positive cells; CD8SP, CD8^+^ simple positive cells; and DP, CD4^+^/CD8^+^ double positive cells). (**B**) Evolution of average percentages of viable cells throughout 10 days of culture. (**C**) Fold expansion of the cells between Days 3 and 7 of culture. Each symbol corresponds to an individual. (**D**) Evolution of average percentages of cells with the CD25^+^FOXP3^+^ phenotype gated on living cells throughout 10 days of culture. (**E**) Evolution of the average percentages of the CD39 marker on CD25^+^FOXP3^+^ subset throughout 10 days of culture. (**F**) Evolution of the average percentages of the CTLA-4 marker on the CD25^+^FOXP3^+^ subset throughout 10 days of culture. The mean + SEM of 14 independent experiments in each condition is shown. * Significant difference when *p* < 0.05. A * above each point condition represents a significant difference for this condition against CD25^+^. A lateral * with connection lines represents significant differences between TGF-β1-treated and untreated conditions for each fraction.

On the day of cell isolation, most of the CD25^neg^ fraction was CD4/CD8 double-positive (DP) and showed a low frequency of CD25 or FOXP3 expression. The phenotype of the CD25^+^ fraction was predominately CD4 simple positive (CD4SP), CD25^+^, and FOXP3^+^ (Figure 1A). Both fractions showed low CD8 simple positive (CD8SP) frequencies (Figure 1A). We examined the cell cultures on Days 3, 7, and 10 post-culture and observed no significant differences in viability (Figure 1B) or cellular proliferation (Figure 1C) among any of the conditions. The CD25^+^ subset proliferated with an average of fourfold expansion (4.06 ± 1.57, mean fold expansion ± SEM) in contrast to the CD25^neg^ subset, either non-treated or TGF-β1-treated (2.90 ± 0.54 and 3.00 ± 0.98, mean fold expansion ± SEM, respectively) (Figure 1C).

The CD25^+^FOXP3^+^ subset phenotype was analyzed. A significant increase in the frequency of the CD25^+^FOXP3^+^ subset in the CD25^neg^ fractions could be observed on the third day after activation. The untreated cells only presented transitory CD25^+^FOXP3^+^ expression, with a peak in expression on Day 3 post-culture, undoubtedly due to cellular activation (Figure 1D and Figure S1 in Supplementary Materials). Conversely, the sustained expression of CD25 and FOXP3 over the 10 days was only observed in the TGF-β1-treated condition (Figure 1D and Figure S1), but was slightly lower in the TGF-β1-treated CD25^neg^ than in the CD25^+^ fraction on Days 7 and 10 of culture (70.69 ± 5.88% for the TGF-β1-treated CD25^neg^ subset and 89.62 ± 3.35% for the CD25^+^ subset on Day 10 of culture, mean ± SEM, *p* = 0.0016, Figure 1D).

Additionally, we analyzed if these treated thymocyte-derived cells might express CD39 or CTLA-4, which are directly associated with FOXP3 expression in nTregs and participate in Tregs’ suppressive function [34,35]. On the day of cell isolation, the expression of CD39 gated on the CD25^+^FOXP3^+^ subset was significantly different between the CD25^+^ fraction (37.64 ± 5.31%, mean ± SEM) and the other conditions (CD25^neg^, 11.54 ± 2.24% and TGF-β1-treated CD25^neg^, 11.69 ± 2.29%, mean ± SEM, Figure 1E). On the one hand, the CD39 expression in the CD25^neg^ condition was surprisingly increased as the culture time increased, regardless of whether the cells were treated or not with TGF-β1, while its expression was decreased in the CD25^+^ fraction (Figure 1E). On the other hand, CTLA-4 was highly expressed in all the conditions on the isolation day (Figure 1F), with a peak in expression on Day 3 post-culture in the CD25^+^ fraction (82.95 ± 3.43%) and TGF-β1-treated CD25^neg^ (74.81 ± 4.83%, mean ± SEM, Figure 1F). Its expression decreased as the culture time increased (CD25^+^, 34.10 ± 15.98%, and TGF-β1-treated CD25^neg^, 43.75 ± 13.53% on Day 10 post-culture, mean ± SEM, Figure 1F).

It has been previously described that TGF-β1 increases the FOXP3 expression and decreases the proliferation of peripheral naïve Tconv cells (CD4^+^CD25^neg^), generating iTregs in the periphery [11]. To verify that TGF-β1 acted equally, we treated the activated-peripheral naïve Tconvs with IL-2 and TGF-β1 as controls (Figure S2A). As expected, the TGF-β1 treatment induced FOXP3 expression in Tconvs (Figure S2A,B) in comparison to untreated Tconvs without reaching the same level as in nTregs (CD25^+^ subset). Moreover, the TGF-β1-treated cells presented a slightly lower proliferation than their untreated counterparts, showing that TGF-β1 treatment modified peripheral Tconvs as previously described (Figure S2C).

Therefore, primary human thymocyte-derived cells, when duly stimulated, could be kept in culture for at least 10 days, and TGF-β1 stimulation induced a high and stable expression of CD25, FOXP3, and CTLA-4 as well as the acquisition of the CD39 marker in the CD25^neg^ subset, as observed in peripheral iTregs.

### 4.2. TGF-β1 Treatment Did Not Induce a Robust Suppressive Function in CD25^neg^ Cells

Since the TGF-β1-treated CD25^neg^ fractions acquired a regulatory-like phenotype constituted of CD25, FOXP3, CTLA-4, and CD39 expression, we studied whether these cells could have developed a suppressive function. Hence, we analyzed the suppressive capacity of the cells on Day 7 post-culture by measuring their ability to block the proliferation of CellTrace Violet (CTVio) cells (allogeneic peripheral blood mononuclear cell (PBMC), Figure S3).

**Figure 2:**
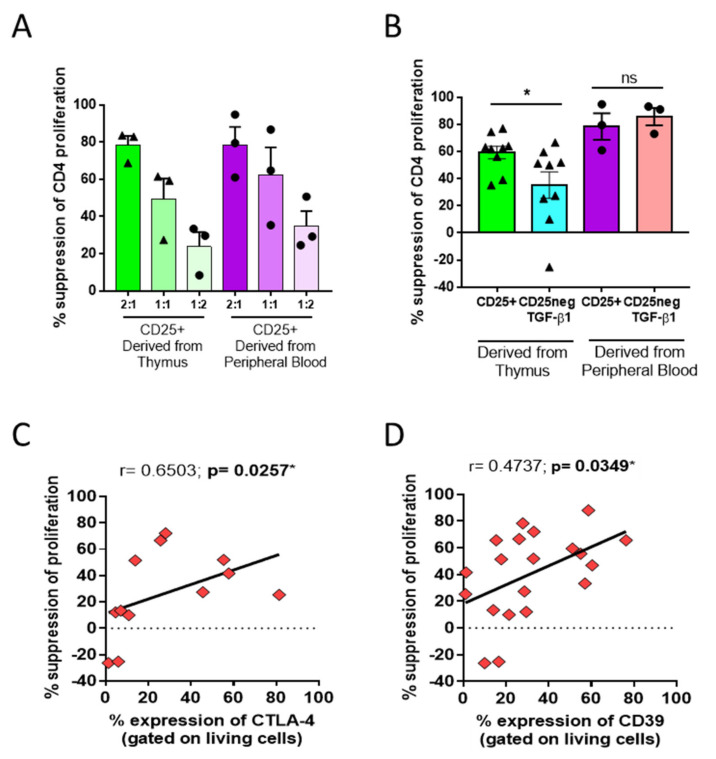
Suppression of CellTrace Violet (CTVio) allogeneic PBMC proliferation by peripheral and thymus-derived cells. (**A**) Percentage of suppression of proliferation of CD4^+^ CTVio cells after co-culture with tCD25^+^ or peripheral CD25^+^ (nTregs) for 7 days, with different ratios of effector cells/CTVio cells. The percentage of suppression was calculated following the division index method. Each symbol corresponds to an individual. (**B**) Percentage of suppression of proliferation of CD4^+^ CTVio cells after co-culture with CD25^+^ and TGF-β1-treated CD25^neg^ derived from the thymus or peripheral blood. Negative values correspond to an increase in proliferation. Each symbol corresponds to an individual. * represents a significant difference for the condition compared to CD25^+^, and ns means non-significant. (**C**) Correlation and linear regression (black line) between the percentage of suppression of CD4^+^ CTVio cells and percentage of CTLA-4 (on CD25^neg^ and TGF-β1-treated CD25^neg^, gated on living cells). (**D**) Correlation and linear regression (black line) between the percentage of suppression of CD4^+^ CTVio cells and percentage of CD39^+^ cells (on CD25^neg^ and TGF-β1-treated CD25^neg^, gated on living cells). Each symbol corresponds to an individual. Correlations were determined by Spearman’s rank correlation, and *r* is the Spearman correlation coefficient. * *p* < 0.05 was considered to be statistically significant.

The thymus-derived CD25^+^ subset limited CD4 CTVio cell proliferation in a dose-dependent manner (Figure 2A). This suppression was similar to that observed in the suppressive assay with nTregs isolated from peripheral blood (Figure 2A). As a control, we showed that naïve TGF-β1-treated CD25^neg^ Tconvs could limit allogenic CD4 proliferation in a manner similar to that observed for nTregs (Figure 2B). However, TGF-β1-treated CD25^neg^ derived from the thymus could only partially limit the proliferation of target cells, and this suppressive function was heterogeneous (Figure 2B). A positive correlation between the frequency of CTLA-4 and CD39 expression and the suppressive function was observed in the thymus-derived cells (CD25^neg^ and CD25^neg^ + TGF-β1) (*p* = 0.0257 and *p* = 0.0349, respectively, Figure 2C,D). Therefore, even though TGF-β1 induced strong FOXP3 expression, thymus-derived CD25^neg^ cells could not acquire a suppressive function similar to that observed from Tconvs, and the suppressive ability was correlated with CTLA-4 and CD39 expression.

### 4.3. Cellular Stability in the Suppressive Assay

**Figure 3:**
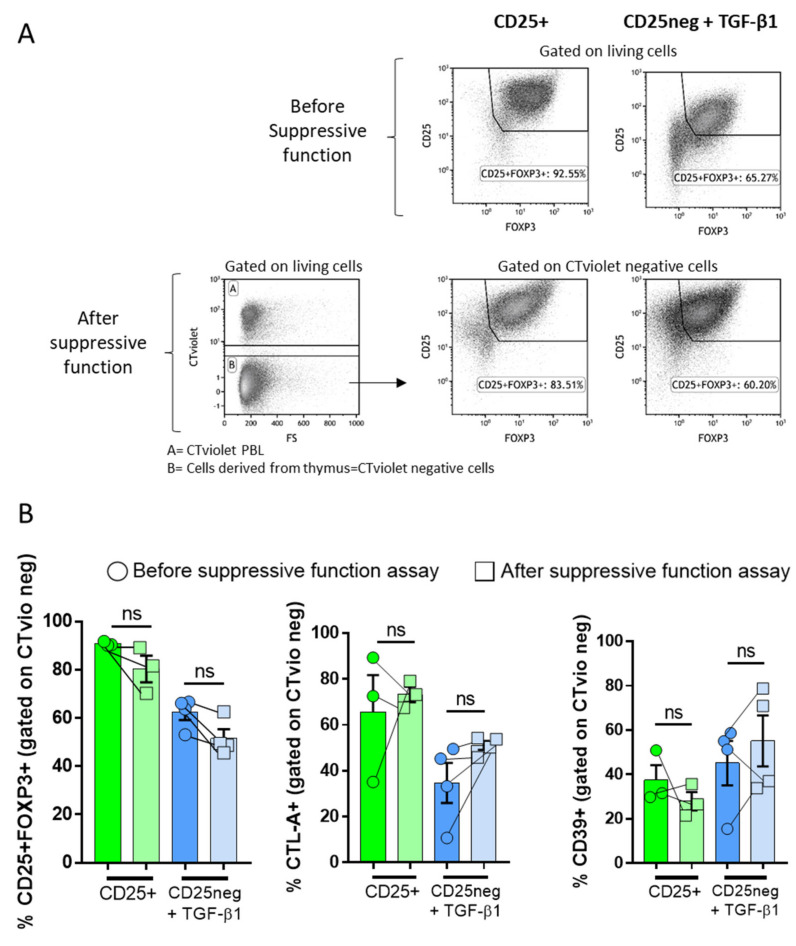
Cell phenotype before and after the suppressive assay. After 7 days of culture, CD25^+^ and TGF-β1-treated CD25^neg^ (effector cells) were co-cultured with CTVio allogenic PBMCs for 3 days, with an effector cell/CTVio cell ratio of 2:1. (**A**) The top panels show dot plots that represent the percentage of CD25/FOXP3 surface markers of each fraction on the day of the suppressive assay setup. The lower panels show dot plots that represent the gating strategy used after the suppressive assay, which mixed thymus-derived cells and CTVio cells. To analyze the thymus-derived cell surface markers, cells were gated on CTVio-negative cells. The dot plots are from a single representative experiment of 3–4 experiments. (**B**) Bar graph representing the frequency of CD25^+^FOXP3^+^ (**left**), CTLA-4^+^ (**middle**), and CD39^+^ (**right**) gated on living CTVio-negative cells before and after the suppressive assay throughout Day 3 of co-culture. Each symbol corresponds to an individual. And ns means non-significant.

We observed a heterogeneous suppressive ability for TGF-β1-treated CD25^neg^ derived from the thymus, which could be linked to phenotypic instability. Therefore, we checked the Treg-associated cellular markers before and after the suppressive assay (Figure 3A). In the co-culture suppressive assay, the cells derived from the thymus were negative for the CTVio signal. Although CD25^+^FOXP3^+^ expression was slightly decreasing at the end of the suppression assay in the CD25^+^ subset, it was still higher than in the TGF-β1-treated CD25^neg^ condition (Figure 3B). On the contrary, the expression of the CD39 and CTLA-4 markers was unchanged in both subsets (Figure 3B). Therefore, the lack of a stable suppressive function of the treated CD25^neg^ cells could not be explained by an unstable expression of suppressive markers during the three days of the co-culture suppressive assay, since no changes in the cell markers were observed.

### 4.4. Ectopic Expression of FOXP3 in CD25^neg^CD8^neg^-Derived Thymus Cells

The CD4SP subset represented most of the cells in all the cell culture conditions, and its proportion increased as the culture time increased, especially in the CD25^neg^ fractions (Figure S4A). However, we observed notable frequencies of CD8SP and CD4^+^/CD8^+^ double-positive cells (DP) (21.55 ± 2.88% and 10.52 ± 1.64%, respectively, mean ± SEM) in the TGF-β1-treated CD25^neg^ subset on Day 10 post-culture, which might explain the lack of solid suppressive function acquisition (Figure S4B,C). Therefore, to reduce the frequencies of CD8SP and DP that could influence the functionality of the stimulated CD25^neg^, we isolated the CD25^neg^CD8^neg^ fraction, and then CD8SP represented 4.63 ± 1.39% (mean ± SEM) of the CD25^neg^CD8^neg^ subset (Table 1). 

**Figure 4:**
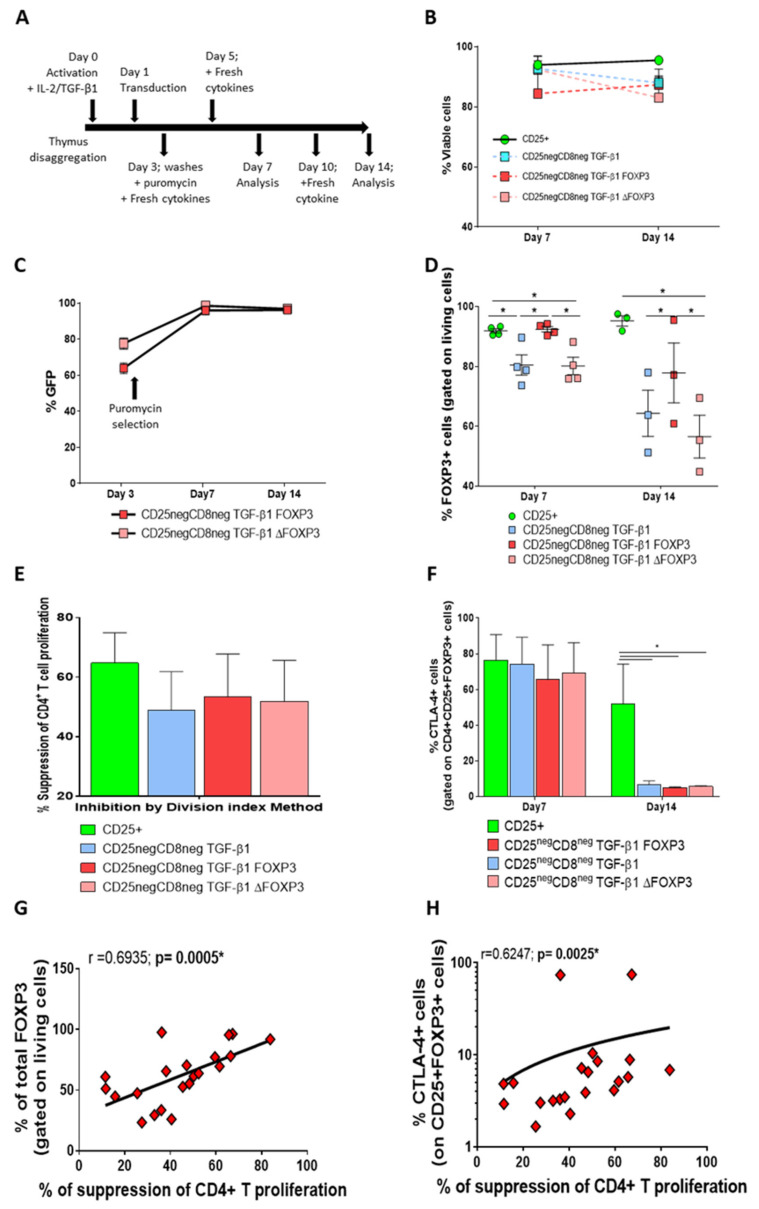
Viability and functionality of the efficiently FOXP3-transduced thymocytes. (**A**) Schematic representation of the experimental protocol, from thymus disaggregation to Days 0–14. The arrows indicate the day and procedure performed at each step. (**B**) The percentage of viable cells for each condition was determined on Days 7 and 14 post-culture by flow cytometry (mean ± SEM of 3–4 independent experiments). (**C**) Analysis of frequencies of transduced cells by flow cytometry after transduction with a vector encoding FOXP3, green fluorescent protein (GFP), and puromycin sequences (mean ± SEM of four independent experiments). The arrow represents the moment of puromycin treatment. (**D**) Percentage of cells expressing intracellular FOXP3, gated on living cells, determined by flow cytometry on Days 7 and 14 post-culture (mean ± SEM of 3–4 independent experiments). (**E**) Percentage of suppression of CD4^+^ CTVio target cell proliferation, calculated by using the division index method (mean ± SEM of three independent experiments). (**F**) Bar graph of CTLA-4 expression on the CD4^+^CD25^+^FOXP3^+^ subset on Days 7 and 14 post-culture by flow cytometry (mean ± SEM of four independent experiments). * *p* < 0.05 was considered to be significant when comparing conditions. (**G**) Correlation between the frequency of suppression of CD4^+^ T CTVio cell proliferation and frequency of total FOXP3^+^ cells (gated on living cells). (**H**) Correlation between the frequency of suppression of the CD4^+^ T CTVio cell proliferation frequency of CTLA-4^+^ (gated on CD4^+^CD25^+^FOXP3^+^). Correlations were determined by Spearman’s rank correlation and considered to be statistically significant, * when *p* < 0.05. Each symbol corresponds to an individual.

With this CD25^neg^CD8^neg^ fraction, we tried to stabilize FOXP3 expression by transducing this subset with MSCV-FOXP3 or MSCV-ΔFOXP3 in combination with IL-2 and TGF-β1 and CD3/CD28 stimulation, as depicted in Figure 4A. The cells were followed over 14 days of culture, with more than 60% of cell viability in all conditions Figure 4B and Figure S5A).

We determined the frequencies of the transduced cells by GFP expression, since the FOXP3-expressing vector also codes for GFP (Figure S5B). On Day 7 and after puromycin treatment, almost all the cells were efficiently expressing GFP (97.60 ± 1.16% for CD25^neg^CD8^neg^FOXP3 and 98.61 ± 0.41% for CD25^neg^CD8^neg^ΔFOXP3, mean ± SEM, Figure 4C and Figure S5B).

As expected, the CD25^+^ fraction presented high levels of FOXP3 (Figure 4D and Figure S5C), and endogenous and exogenous FOXP3 protein were found in the nucleus in the CD25^+^ and transduced cells, where it carried out its function (Figure S5D). On Day 7 post-culture, the FOXP3 levels observed in CD25^+^ were similar to those in the TGF-β1-treated, FOXP3-transduced CD25^neg^CD8^neg^ cells (91.88 ± 0.70% and 92.39 ± 0.89%, respectively, mean ± SEM, Figure 4D). On Day 14 post-culture, the TGF-β1-treated, FOXP3-transduced CD25^neg^CD8^neg^ subsets still expressed FOXP3 at a high level (77.83 ± 9.99%, mean ± SEM), but did not exhibit the homogeneous FOXP3 levels observed in the CD25^+^ fraction (95.21 ± 1.71%, mean ± SEM). Therefore, a reduction in FOXP3 expression as the culture time increased was observed, although 96.24 ± 1.16% (mean ± SEM) of the TGF-β1-treated, FOXP3-transduced CD25^neg^CD8^neg^ cells expressed GFP on Day 14 post-transduction (Figure 4C). In summary, thymocyte-derived cells could be transduced with lentiviral vectors efficiently without affecting their viability, but ectopic FOXP3 expression was slightly reduced and was believed to be unstable as the culture time increased.

We tested whether the overexpression of FOXP3 in the TGF-β1-treated CD25^neg^CD8^neg^ subset could generate cells with a stable suppressive ability. Stimulated transduced or non-transduced TGF-β1-treated CD25^neg^CD8^neg^ subsets showed a basal suppressive ability over CD4^+^ T cell proliferation, but this suppressive ability was lower than that for the CD25^+^ subset (Figure 4E). Interestingly, no significant differences were observed in the suppressive function between the FOXP3- and ΔFOXP3-transduced, TGF-β1-treated CD25^neg^CD8^neg^ conditions, showing that the overexpression of exogenous FOXP3 did not induce a better suppressive ability (Figure 4E). We followed the CTLA-4 expression over the culture time, clearly observing high CTLA-4 expression gated on CD4^+^CD25^+^FOXP3^+^ in all the conditions at Day 7 post-culture (Figure 4F). However, this expression was significantly reduced after 14 days of culture in all the CD25^neg^CD8^neg^ conditions compared with the CD25^+^ subset (Figure 4F). Moreover, we found a positive correlation between the frequencies of suppression of CD4^+^ CTVio cell proliferation and the frequencies of the total FOXP3^+^-expressing cells (Figure 4G), as well as with the frequency of the CTLA-4^+^-expressing cells (Figure 4H), when analyzing all the conditions (CD25^+^, CD25^neg^CD8^neg^ + TGF-β1, transduced or non-transduced, Figure 4H).

Therefore, the ectopic expression of FOXP3 in primary thymic TGF-β1-treated CD25^neg^CD8^neg^ cells is feasible with high viability. However, the induced FOXP3 and CTLA-4 expression, which were reduced as the culture time increased, did not generate a robust and homogeneous suppressive function.

### 4.5. TGF-β1-Stimulated Cells Produced Higher Levels of Anti-Inflammatory and Proinflammatory Cytokines Than the CD25^+^ Subset

**Figure 5:**
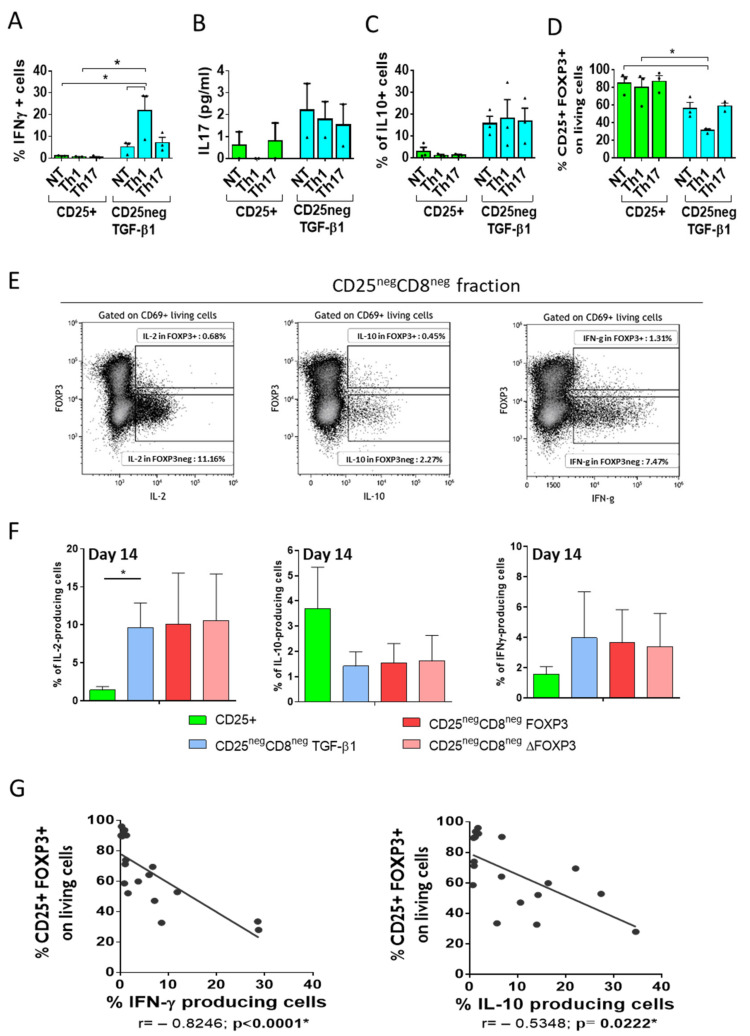
Cytokine expression of thymus-derived cells after restimulation. On Day 7, the TGF-β1-treated CD25^neg^ and CD25^+^ fractions were restimulated with anti-CD3/CD28 beads (NT) as the control condition or were restimulated with anti-CD3/CD28 beads under Th1- or Th17-polarizing conditions. (**A**) Frequencies of IFN-γ-producing cells, measured by flow cytometry. The mean + SEM of three independent experiments are shown. (**B**) IL-17 (pg/mL) levels from the supernatant were measured by ELISA. The mean + SEM of two independent experiments are shown. (**C**) Frequencies of IL-10-producing cells were measured by flow cytometry. The mean + SEM of three independent experiments are shown. (**D**) Percentages of CD25^+^FOXP3^+^ cells gated on living cells were measured by flow cytometry. The mean + SEM of three independent experiments are shown. * *p* < 0.05 was considered to be significant when comparing conditions. We also analyzed cytokine production in cells restimulated without a polarizing environment. (**E**) Example of the gating strategy for selecting cytokine-producing cells in the CD25^neg^CD8^neg^ fraction, according to the expression of FOXP3. Intracellular IL-2-, IL-10-, or IFN-γ-producing cells in FOXP3^+^ and FOXP3^neg^ subsets were assessed by flow cytometry. (**F**) We then calculated the frequencies of cytokines detected in the total cells (FOXP3^+^ and FOXP3^neg^ subsets) (mean ± SEM of 3–4 independent experiments). * *p* < 0.05 was considered to be significant when comparing conditions. (**G**) Correlations between frequencies of the CD25^+^FOXP3^+^ cells gated on living cells, as well as the frequency of IFN-γ-producing cells (left) and frequency of IL-10-producing cells (right), are represented. Correlations were determined by Spearman’s rank correlation and considered statistically significant when *p* < 0.05. Each symbol corresponds to an individual.

One of the most valuable nTreg features demonstrated regarding their use in immunotherapy is their stability under inflammatory conditions [5,36]. Due to the phenotypic instability of the stimulated cells, we examined transduced and non-transduced TGF-β1-treated CD25^neg^ stability under inflammatory environments. The CD25^+^ fraction restimulated under Th1- or Th17-polarizing conditions showed almost no IFN-γ- or IL-10-producing cells and no secretion of IL-17 compared with the non-treated (NT) condition. Therefore, tTregs were not susceptible to change under proinflammatory conditions, as expected (Figure 5A). Conversely, the TGF-β1-treated CD25^neg^ showed a significant increase in the frequencies of IFN-γ-producing cells in the Th1-polarizing condition compared with the CD25^+^ fraction (Figure 5A). The same pattern was detected in the production of IL-17 and the frequencies of IL-10-producing cells (Figure 5B,C). Moreover, the frequencies of CD25^+^FOXP3^+^ cells in the CD25^+^ subset were not affected when the cells were restimulated under Th1- or Th17-polarizing conditions, whereas the TGF-β1-treated CD25^neg^ subset showed a decrease in CD25^+^FOXP3^+^ frequency when cultured under the Th1-polarizing condition (Figure 5D).

In addition, we quantified the expression of intracellular IL-2-, IL-10-, or IFN-γ in our transduced, TGF-β1-treated CD25^neg^CD8^neg^ cells when they were restimulated, but without a proinflammatory environment, compared with the restimulated CD25^+^ subset. The intracellular cytokine expression was different between the FOXP3^+^ and FOXP3^neg^ subsets, being lower in the former, showing that FOXP3^+^ cells express fewer cytokines (Figure 5E). Moreover, gated on total living cells, higher frequencies of IL-2- and IFN-γ-producing cells, and lower frequencies of IL-10-producing cells were observed in all the CD25^neg^CD8^neg^ conditions compared with those in the CD25^+^ subset, even though the differences were not significant (Figure 5F).

Finally, negative associations between the frequencies of the CD25^+^FOXP3^+^ population and IFN-γ- or IL-10-producing cells were observed when analyzing all the non-CD25^+^ conditions (Spearman *r* = −0.8246, *p* < 0.0001, and Spearman *r* = −0.5348, *p* = 0.0222, respectively; Figure 5G). Therefore, the lower the CD25^+^FOXP3^+^ expression, the more anti-inflammatory and proinflammatory cytokines that were produced.

In summary, the CD25^+^ fraction derived from the thymus showed evident stability in CD25 and FOXP3 expression and in cytokine production, despite being under Th1 and Th17 environments, which was not the case for all the CD25^neg^CD8^neg^ subsets. These results suggest that the cellular instability of FOXP3 and CTL-4 expression, as well as the susceptibility to producing proinflammatory cytokines after stimulation in TGF-β1-treated or FOXP3-transduced CD25^neg^CD8^neg^ subsets, may cause low suppressive capacities in these cells. 

## 5. Discussion

In this study, we showed that the overexpression of the principal nTreg transcription factor, FOXP3, by TGF-β1 treatment or by ectopic FOXP3 expression was insufficient for conferring a stable phenotype and function to the CD25^neg^-derived thymocytes [37,38]. All the results were summarized in Table S2.

To induce suppressive iTregs from the periphery, various factors are essential, such as IL-2, TCR/CD28 engagement, and TGF-β1 [38]. It is known that, due to their natural plasticity, pTregs and iTregs from peripheral naïve CD4^+^ T cells can lose their FOXP3 expression under certain circumstances [23,39]. However, this instability could be implicated in the Treg natural selection of self and non-self in the thymus, which is related to the TCR signal strength [40]. Therefore, the physiological function of Treg instability is a complex phenomenon that is not fully understood. Here, the TGF-β1-treated CD25^neg^ and TGF-β1-treated FOXP3-transduced cells permitted the acquisition of robust FOXP3 expression, similar to that observed in the peripheral Tconvs. However, the expression of FOXP3 and other Treg-related markers was not stable enough and was slightly reduced as the culture time increased. It has been previously demonstrated that the expression of FOXP3 alone is not sufficient, even in the thymus [41]. In fact, demethylation of the FOXP3 promoter and its subsequent expression were not associated with a functional Treg phenotype [42]. Extrinsic factors, such as the IL-2/IL-2 receptor axis and other cytokines, are pivotal to maintaining Treg cell integrity and functionality [43,44]. CD25^neg^FOXP3^low^ and CD25^+^ FOXP3^neg^ progenitors can both be differentiated to CD25^+^ FOXP3^+^ with high efficiency when a high concentration of IL-2 is used [25]. It has been described that IL-2 is essential for regulating and maintaining FOXP3 expression [45]. In this study, we tried to differentiate the CD25^neg^ subset with a high concentration of IL-2 and TGF-β1, and the cells presented a clear expression of FOXP3 without acquiring a stable and robust suppressive function.

CTLA-4 has also been shown to regulate the early development of self-reactive T cells in the thymus. It plays a key role in the central tolerance and the development of conventional and regulatory T cells [46]. Therefore, this might explain why the reduction in CTLA-4 and FOXP3 expression in the non-CD25^+^ subsets correlated with a lower suppressive function, suggesting that phenotypic instability could be related to functional instability. Supporting these ideas, Ohkura et al. demonstrated the need for an elevated demethylation state of the FOXP3 Treg-specific demethylated region as well as demethylation in other Treg-associated genes, such as *CTLA-4*, to acquire lineage stability and full suppressive activity [41,47,48,49]. Therefore, further studies on the differences in the methylation states between tTreg and TGF-β1-treated thymocytes would help us understand the impact of the epigenetic status of these cells on their function.

Another hypothesis to explain the instability of generated cells is that, although exogenous FOXP3 expression was forced in the CD25^neg^ thymocytes, these cells could lack regulatory and post-transcriptional factors essential for Treg development and maintenance, such as the sterile motif histidine–aspartate domain containing protein 1 [50] or the Forkhead box P1 [51], which stabilizes FOXP3 expression in Tregs, showing the importance of the intrinsic cellular gene environment. It has previously been discovered and well-reviewed that a degradation mechanism for FOXP3 or cytoplasmic translocation could happen [52,53]. Indeed, activated CD4^+^ Tconvs showed a FOXP3 cytoplasmic localization, which might explain why the transitory FOXP3 expression after cellular activation was not followed by a robust suppressive ability. In our work, we demonstrated that FOXP3 expression was localized in the nucleus, where FOXP3 carries out its function.

The direct deacetylation, phosphorylation, and especially ubiquitylation of FOXP3 also play essential roles in regulating Treg cell functions. It has been demonstrated that the deubiquitinase USP7 was highly expressed in Treg cells, and its absence was associated with diminished FOXP3 expression and a subsequent decrease in Treg-cell-mediated suppression in vitro [54]. The U-box domain type E3 ubiquitin ligase STUB1 was also demonstrated to degrade FOXP3, which subsequently provoked a reduction in the expression of Treg cell-associated genes, the upregulation of IL-2 and IFN-γ expression, and finally, the disruption of the suppressive function of Treg cells [55]. Therefore, the possible FOXP3 degradation could explain the reduction in CTLA-4 and the increase in IL-2 and IFN-γ expression observed in our work, since it has been previously demonstrated that FOXP3 could directly act on the *CTLA-4*, *IL-2,* and *IFN-γ* genes through direct chromatin remodeling by histone acetylation and deacetylation [56,57,58]

Consequently, cellular activation associated with the overexpression of exogenous FOXP3 in thymocytes, as well as TGF-β1/IL-2 treatment, was necessary to increase FOXP3 expression, but not sufficient to convert CD25^neg^CD8^neg^ derived from the thymus into Treg-like cells. This lack of capability could be related to the immature stage of the cells, the absence of a positive gene environment, FOXP3 degradation, or the epigenetic status of the stimulated cells. Hence, understanding the whole mechanism of Treg generation could unlock ways to manipulate the epigenetic status or culture conditions to enable the cells’ use in therapeutic approaches in the future.

## Figures and Tables

**Table 1:** Frequencies of viability and cellular markers on the day of cell isolation. Frequencies of living cells, CD4 simple positive (SP), CD8SP, Treg (CD25^+^FOXP3^+^ gated on CD4 SP), CD25^+^FOXP3^+^ (gated on living cells), CTLA-4 (gated on CD25^+^FOXP3^+^), and CD39 (gated on CD25^+^FOXP3^+^) were obtained by flow cytometry analysis on the three different subsets obtained the day of thymus-derived cell isolation (total thymocytes, CD25^+^ and CD25^neg^CD8^neg^ fractions). The CD25^+^ subset represents the tTregs. The means between the CD25^+^ and thymocyte values and between the CD25^+^ and CD25^neg^CD8^neg^ values are compared, where *p* < 0.05 is considered to be significant when comparing conditions. ^(a)^ Frequency, mean ± SEM. ^(b)^ The statistical comparisons between conditions were performed using one-way ANOVA followed by Bonferroni’s multiple comparison test.

	Cellular Subsets Analyzed ^(a)^			Statistic Comparison ^(b)^	
	Total Thymocytes (%)	CD25^+^ Subset (%)	CD25^neg^CD8^neg^ Subset (%)	*p* CD25^+^ vs. Thymocytes	*p* CD25^+^ vs. CD25^neg^CD8^neg^
**Living cells**	69.81 ± 4.99	83.22 ± 2.29	81.86 ± 3.40	0.1494	>0.999
CD4 SP	37.51 ± 4.44	69.62 ± 2.47	59.97 ± 6.41	0.0010	>0.999
CD8 SP	12.76 ± 2.47	2.67 ± 0.65	4.63 ± 1.39	0.0643	0.6858
tTreg (CD25^+^FOXP3^+^)	11.59 ± 0.91	82.38 ± 2.69	4.18 ± 0.96	0.0002	0.0001
CD25^+^FOXP3^+^	5.38 ± 0.30	77.44 ± 3.74	2.65 ± 0.46	0.0004	0.0004
CTLA-4	64.96 ± 5.86	77.87 ± 3.47	56.83 ± 9.03	0.1863	0.2215
CD39	23.39 ± 3.88	36.70 ± 7.96	11.13 ± 2.19	0.5334	0.2803

## Data Availability

Not applicable.

## Supplementary Materials

The following are available online at https://www.mdpi.com/article/10.3390/biomedicines9050461/s1. Figure S1: Dot plots of viability and CD25^+^FOXP3^+^ phenotype at Day 7 of the culture. Figure S2: Generation of iTreg from peripheral Tconv. Figure S3: Suppression of CTVio target cell (allogenic PBMC) proliferation. Figure S4: Frequencies of CD4 and CD8 expression in thymus-derived subsets throughout cell culture. Figure S5: Viability, GFP expression and FOXP3 frequency of transduced CD25^neg^CD8^neg^ thymocytes. Table S1: Antibodies for cell surface and intracellular staining. Table S2: Summary of the results.
