# Supplementary material for: Ectopic FOXP3 Expression in Combination with TGF-β1 and IL-2 Stimulation Generates Limited Suppressive Function in Human Primary Activated Thymocytes Ex Vivo
Source: Biomedicines. 2021 Apr 23;9(5):461. doi: 10.3390/biomedicines9050461 (PMC8146103; doi:10.3390/biomedicines9050461)
Supplement: Supplementary file 1 [file biomedicines-09-00461-s001.zip › biomedicines-1186831-supplementary.pptx]

## Slide 1
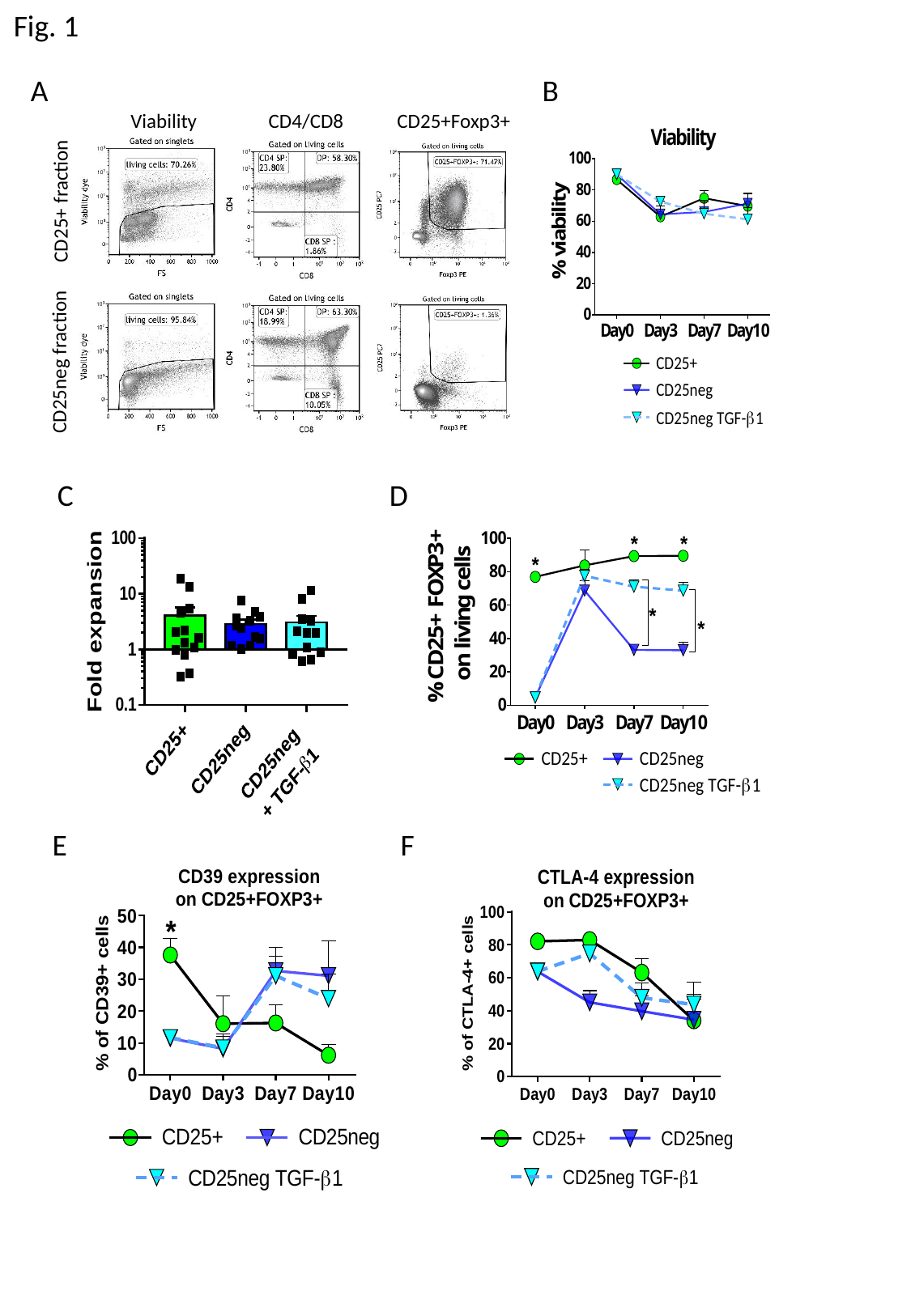

Fig. 1
A
B
CD25+Foxp3+
Viability
CD4/CD8
CD25+ fraction
CD25neg fraction
C
D
E
F

## Slide 2
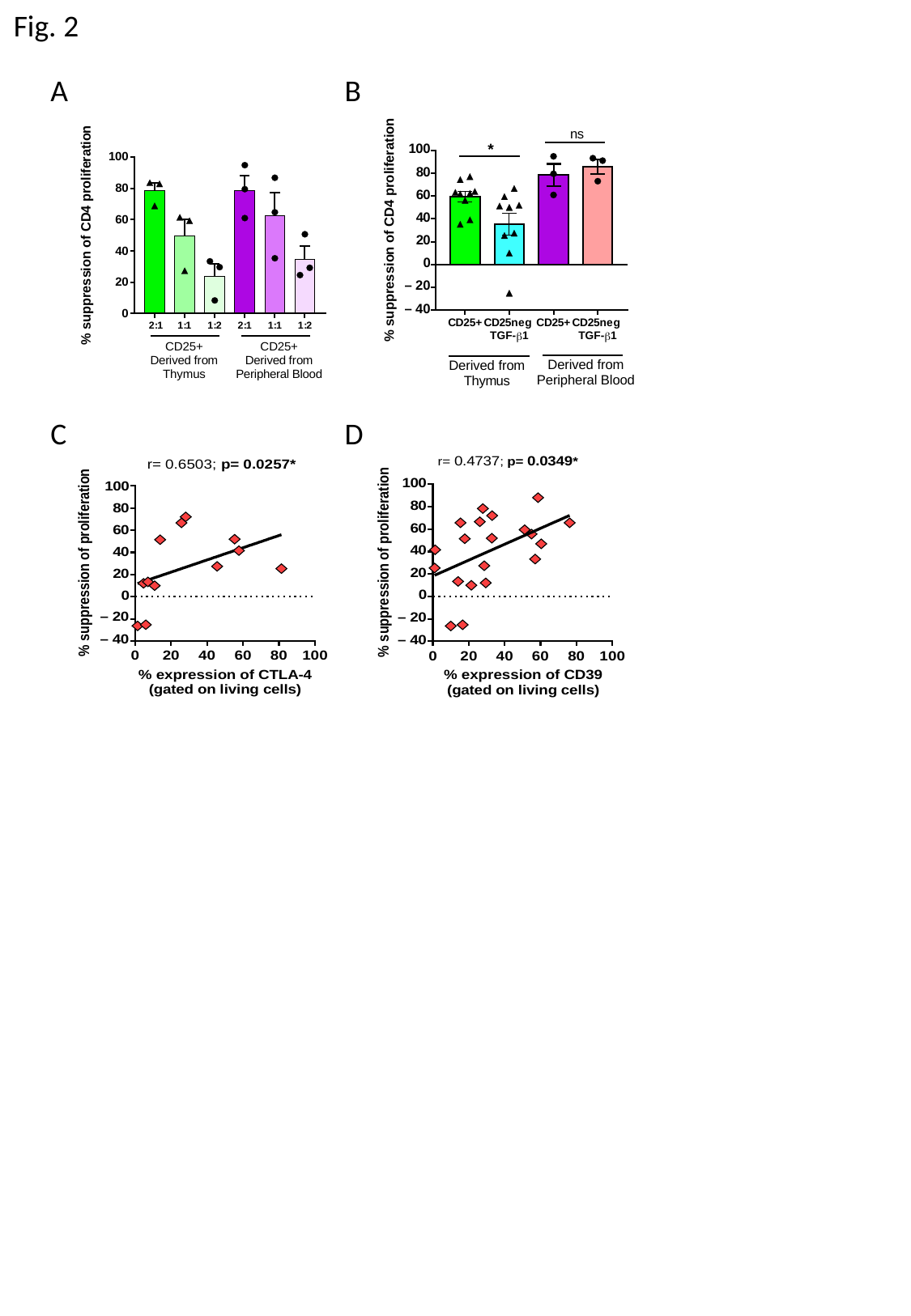

Fig. 2
A
B
C
D

## Slide 3
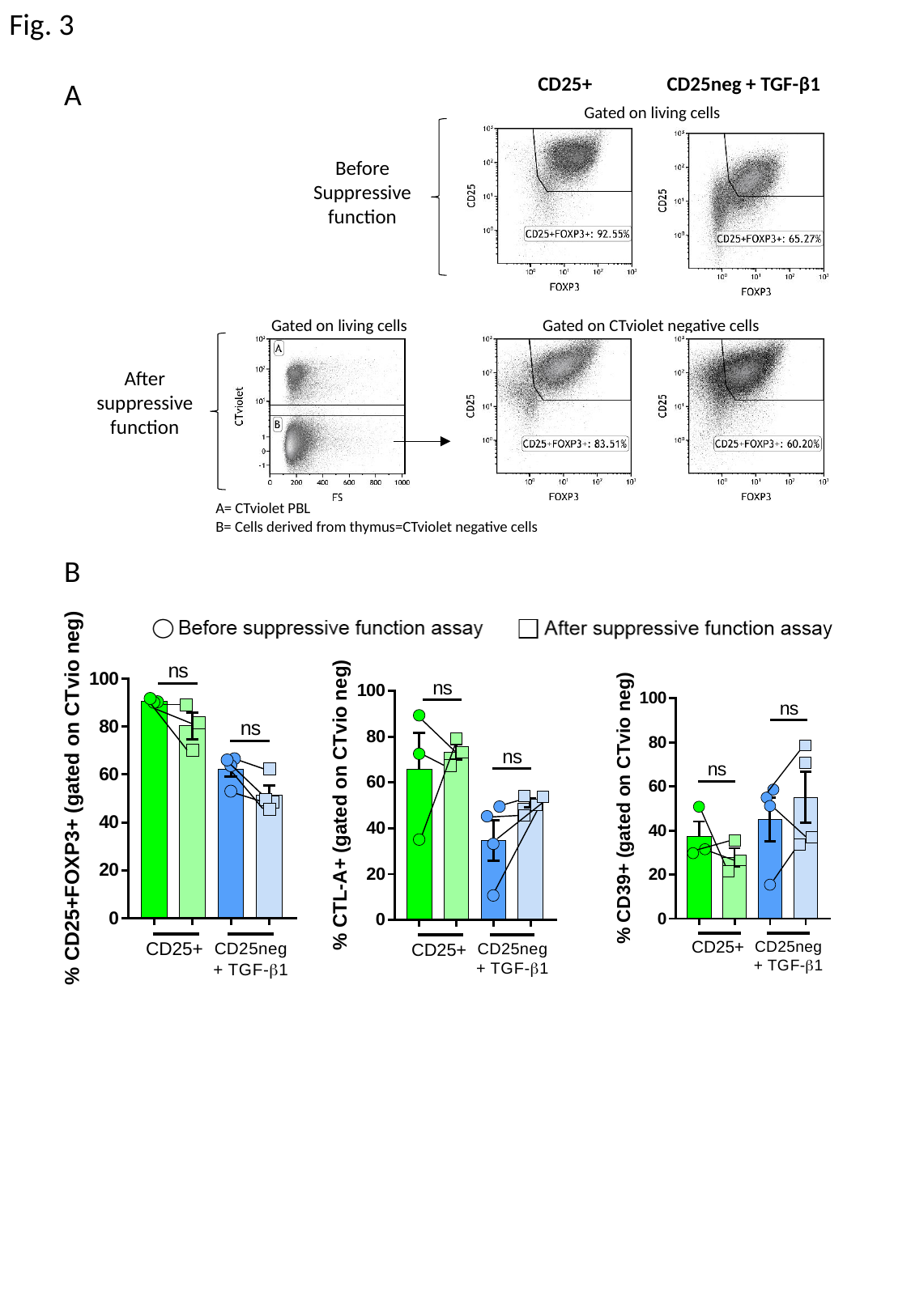

Fig. 3
CD25+
CD25neg + TGF-β1
A
Gated on living cells
Before Suppressive function
Gated on CTviolet negative cells
Gated on living cells
After suppressive function
A= CTviolet PBL
B= Cells derived from thymus=CTviolet negative cells
B

## Slide 4
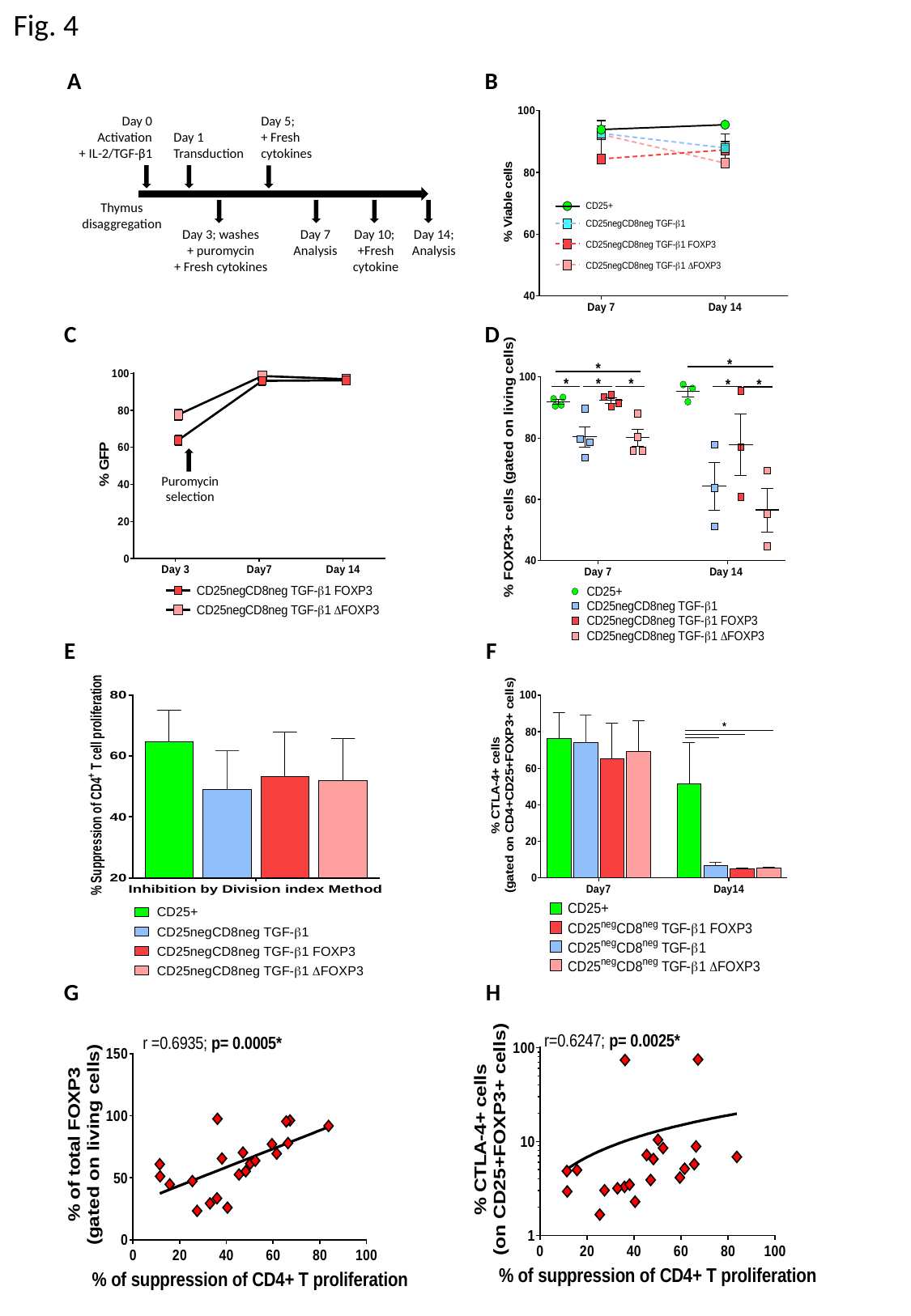

Fig. 4
A
B
Day 5;
+ Fresh cytokines
Day 0 Activation
+ IL-2/TGF-β1
Day 1
Transduction
Thymus disaggregation
Day 7
Analysis
Day 10;
+Fresh cytokine
Day 14;
Analysis
Day 3; washes
+ puromycin
+ Fresh cytokines
C
D
Puromycin
selection
E
F
G
H

## Slide 5
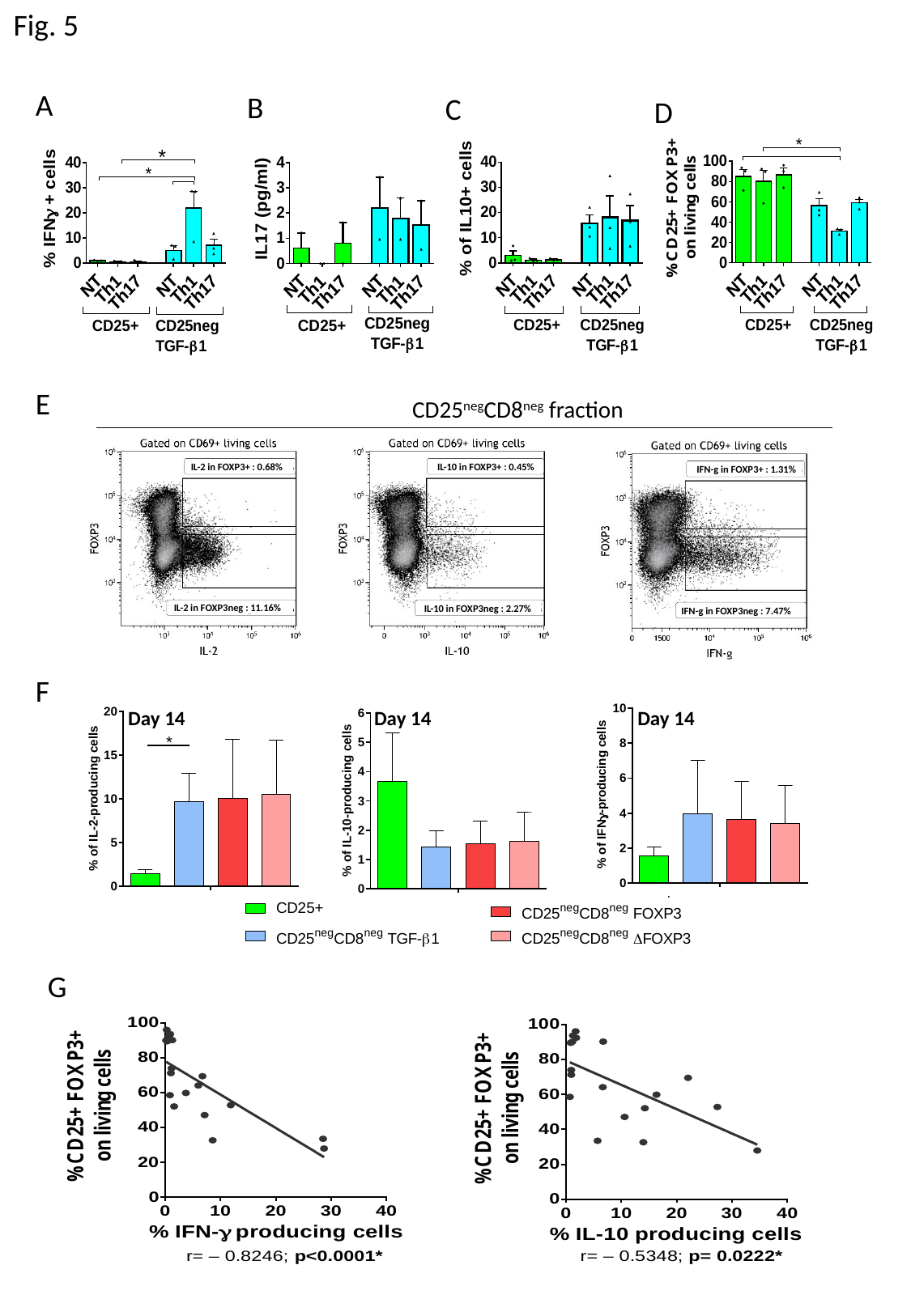

Fig. 5
A
B
C
D
E
CD25negCD8neg fraction
IL-2 in FOXP3+ : 0.68%
IL-10 in FOXP3+ : 0.45%
IFN-g in FOXP3+ : 1.31%
IL-2 in FOXP3neg : 11.16%
IL-10 in FOXP3neg : 2.27%
IFN-g in FOXP3neg : 7.47%
F
Day 14
Day 14
Day 14
G

## Slide 6
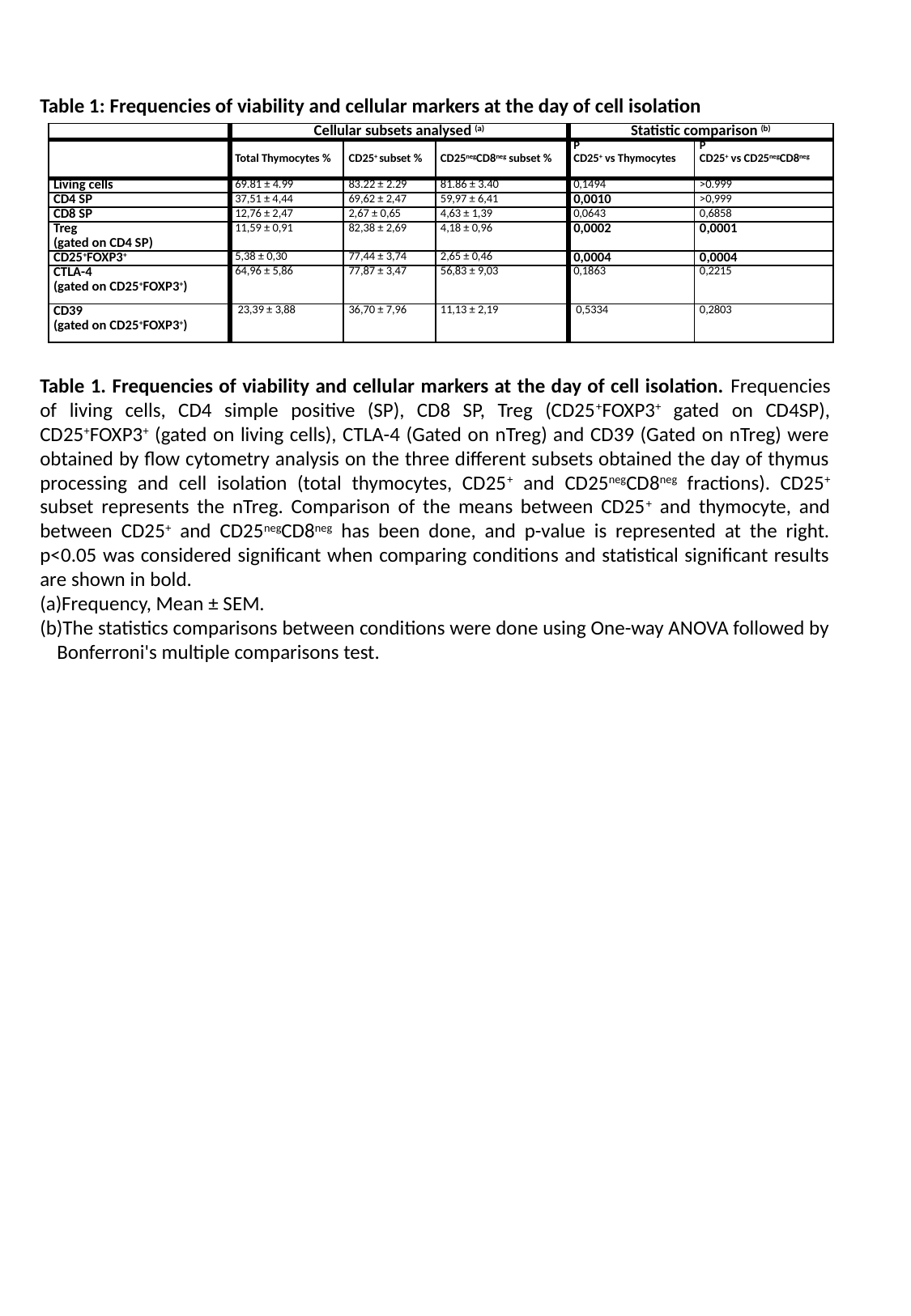

Table 1: Frequencies of viability and cellular markers at the day of cell isolation
| | Cellular subsets analysed (a) | | | Statistic comparison (b) | |
| --- | --- | --- | --- | --- | --- |
| | Total Thymocytes % | CD25+ subset % | CD25negCD8neg subset % | P CD25+ vs Thymocytes | P CD25+ vs CD25negCD8neg |
| Living cells | 69.81 ± 4.99 | 83.22 ± 2.29 | 81.86 ± 3.40 | 0,1494 | >0.999 |
| CD4 SP | 37,51 ± 4,44 | 69,62 ± 2,47 | 59,97 ± 6,41 | 0,0010 | >0,999 |
| CD8 SP | 12,76 ± 2,47 | 2,67 ± 0,65 | 4,63 ± 1,39 | 0,0643 | 0,6858 |
| Treg (gated on CD4 SP) | 11,59 ± 0,91 | 82,38 ± 2,69 | 4,18 ± 0,96 | 0,0002 | 0,0001 |
| CD25+FOXP3+ | 5,38 ± 0,30 | 77,44 ± 3,74 | 2,65 ± 0,46 | 0,0004 | 0,0004 |
| CTLA-4 (gated on CD25+FOXP3+) | 64,96 ± 5,86 | 77,87 ± 3,47 | 56,83 ± 9,03 | 0,1863 | 0,2215 |
| CD39 (gated on CD25+FOXP3+) | 23,39 ± 3,88 | 36,70 ± 7,96 | 11,13 ± 2,19 | 0,5334 | 0,2803 |
Table 1. Frequencies of viability and cellular markers at the day of cell isolation. Frequencies of living cells, CD4 simple positive (SP), CD8 SP, Treg (CD25+FOXP3+ gated on CD4SP), CD25+FOXP3+ (gated on living cells), CTLA-4 (Gated on nTreg) and CD39 (Gated on nTreg) were obtained by flow cytometry analysis on the three different subsets obtained the day of thymus processing and cell isolation (total thymocytes, CD25+ and CD25negCD8neg fractions). CD25+ subset represents the nTreg. Comparison of the means between CD25+ and thymocyte, and between CD25+ and CD25negCD8neg has been done, and p-value is represented at the right. p<0.05 was considered significant when comparing conditions and statistical significant results are shown in bold.
Frequency, Mean ± SEM.
The statistics comparisons between conditions were done using One-way ANOVA followed by Bonferroni's multiple comparisons test.
